# Female levator ani muscle damage assessment in supine and upright position

**DOI:** 10.1038/s41598-025-01266-x

**Published:** 2025-05-10

**Authors:** Irina de Alba Alvarez, Annemarie van der Steen, Anique T.M. Grob, Frieda van den Noort

**Affiliations:** 1 Multi Modality Medical Imaging (M3I), TechMed Centre, University of Twente, Drienerlolaan 5, 7522 NB Enschede, The Netherlands; 2 Department of Gynecology, Ziekenhuisgroep Twente, Hengelo, The Netherlands

**Keywords:** Levator Ani muscle, Pelvic organ prolapse, Magnetic resonance imaging, Iliococcygeus, Medical imaging, Urogenital diseases, Biomedical engineering

## Abstract

There is damage in the pelvic floor only visible in upright imaging. This damage have not been yet studied. The aim of this research is to assess the difference in levator ani muscle (LAM) damage in supine and upright position by means of MRI scans. Sixty-four patients with minimum stage 2 prolapse of the anterior vaginal wall or uterus, without previous pelvic organ prolapse (POP) surgery were scanned in a MR scanner in supine and upright position. Damage to the pubococcygeus muscle (PCM) and the iliococcygeal muscle (ICM) was scored as none, minor or major. For PCM, a previously established protocol was used. For ICM, a protocol for damage assessment was established and validated in this study, by determining the interclass correlation coefficient (ICC). The new ICM assessment protocol was established with ICC values of 0.68 (0.57–0.77) for supine and 0.81 (0.74–0.86) for upright assessment. 6.3% major ICM damage was found in supine vs. 51.6% in upright position with a significant difference of p < 0.001 for the sign-test. There was an underestimation of ICM damage in 59% of the cases in supine position. PCM damage scoring was not feasible on upright MRI’s and therefore only assessed on supine scans. In our POP population we found 53.1% of the women with major damage to either the PCM or ICM and 32.8% with major damage in both, leading to a total of 85.9% of women with major damage to at least one structure. There is a significant difference in LAM damage assessment between supine and upright position. Supine imaging leads to a severe underestimation of ICM damage while for the PCM supine damage assessment remains superior.

## Introduction

Pelvic Floor Disorders (PFDs), including urinary and fecal incontinence, constipation and pelvic organ prolapse (POP) are common conditions in women^1-3^. PFD prevalence has been reported in up to 25% of the female population and has a considerable negative impact on the quality of life^3^. Previous studies provide evidence to support the hypothesis that levator ani muscle (LAM) damage is correlated to PFDs^4-6^.

Damage has been reported in several parts of the LAM, more specifically the iliococcygeus muscle (ICM) and the pubococcygeus muscle (PCM)^4,7-14^.Detection of the damage can be done by means of medical imaging and palpation^15-17^. Both Magnetic Resonance Imaging (MRI) and Ultrasound (US)^10,18^ provide detailed images of the pelvic musculature. Several grading systems exist to grade the level of levator ani muscle injury, all executed with the patient in supine position^10,13,18^. We found, however, no such system for the ICM. While adequate and complete identification of LAM injuries is needed to improve understanding of PFD pathophysiology.

Recent studies report significant differences in the extent of prolapse and LAM shape when patients are assessed in upright as compared to supine position^19-21^. Therefore, we hypothesize that LAM injury will appear differently in supine and upright position. This could lead to an over- or underestimation of the injuries and therefore to an incomplete understanding of the pathophysiology of PFDs. In this study we aim to develop a ICM injury scoring system and assess consecutive supine and upright MRI scans of patients with POP to determine differences in LAM injury.

## Materials and methods

### Population

This is a secondary analysis of patients with POP recruited from a prospective MRI study including patients prior to and after prolapse surgery. In this study the pre-operative MRI scans of the patients were analysed. The patients were recruited from the gynaecology department of our local hospital. The study was approved by the medical ethics committee (medical ethics committee CMO Regio Arnhem Nijmegen NL79717.091.21) and all patients gave written informed consent. The methods were performed in accordance of the guidelines and regulations of the ethics committee. All women were 18 years or older and had a minimum stage 2 prolapse of the anterior vaginal wall or uterus (based on the Pelvic Organ Prolapse Quantification (POP-Q)^22^, without previous POP surgery. Women were excluded if they were unable to stand for 20 min without assistance, were not eligible to undergo an MR scan in response to a MR safety checklist or had a jeans size ≥ 52 (EU) or 22 (USA), because of the limited MR coil circumference.

## MRI acquisition

MR scans were acquired with the women in supine and upright position. Participants were asked not to drink 1 h prior to the scan and to empty their bladder within 15 min before the scan. A tiltable 0.25 T MR scanner (G-Scan Brio; Esaote S.p.A., Genoa, Italy) was used for MR acquisition, with a dedicated multichannel spine coil. A 3D balanced steady state free precession (bSSFP) sequence was used using the following acquisition parameters: echo time [TE] = 4 ms, repetition time [TR] = 8 ms, voxel resolution 0.49 × 0.49 × 0.49 mm³ and field of view of 250 × 250 × 160 mm³ and an acquisition matrix 124 × 124 × 100. Images were acquired with the patient in supine and upright position consecutively, resulting in a limited time (< 10 min) between scans, thus reducing the influence of variables like bladder and rectal filling.

## Injury grading

LAM damage was graded in supine and upright position separately for the PCM and the ICM in axial, coronal and sagittal axis. The grading of the PCM was done using a previously established protocol by Kearney et al.^10^, providing a score from 0 to 3 for both the left and right side of the muscle. Subsequently, the score of both sides were summed, which resulted in a range from 0 to 6 and is categorized as: 0 = normal/no damage, 1–3 = minor damage (expect for a unilateral score of “3”, which is labelled “major”), 4–6 = major damage.

Since a standardized grading system for the ICM is lacking, a new grading protocol was established, based on ICM parameters described in previous literature. Finetuning of this new protocol was done using supine and upright MRI data from a different study^23^. Inter-observer agreement was assessed on the data presented in this manuscript.

**Fig. 1:**
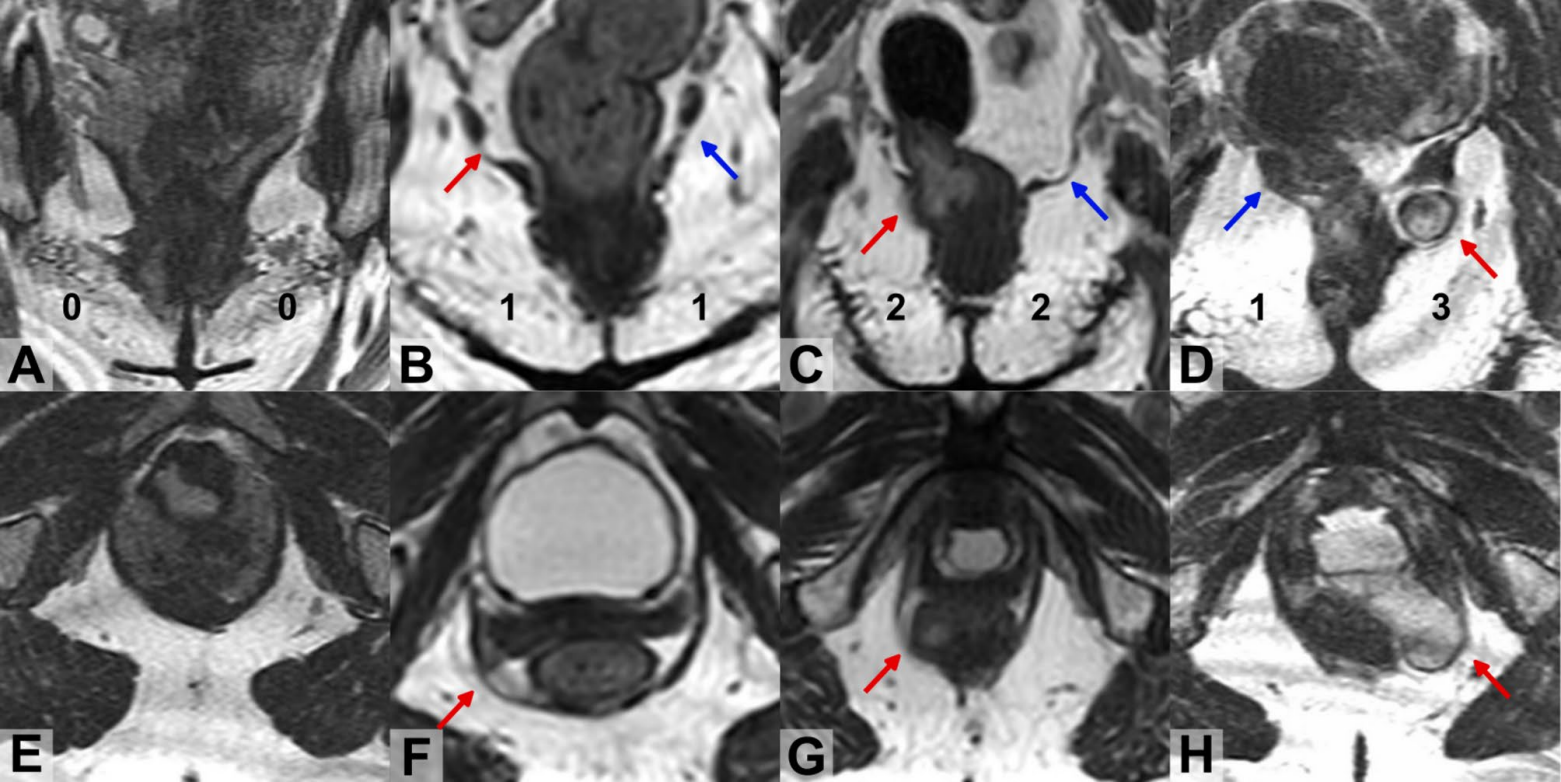
Examples of the appearance of the iliococcygeus muscle damage assessing scoring in coronal (A, B,C, D) and axial (E, F,G, H) MR images. **A** and E represents the normal shape of the muscle; **B** and F showed a woman with minor damage. **C** and G showed a woman with major damage. **D** and **H** showed a woman with major damage with hernia in the left side. Blue and red arrows point ICM damage in coronal view. In axial view, the damage pointed by the red arrow corresponds to the same damage pointed by the red arrow on coronal view.

A score of “0” meant there was either no visible damage to the ICM, or only generalized ballooning without muscle thinning, a result from pressure to the pelvic floor, or a diffuse shape, i.e. small gaps^24,25^,. A score of “1” was given if there was thinning or larger disruptions in the muscle^9,11,12,26^. A score of “2” was used when there was noticeable focal bulging along with thinning or eventration, but the depth was less than 10 mm^9,12,14,27-29^. Lastly, a score of “3” was given for ICM hernias, which is a focal bulging with thinning or eventration deeper than 10 mm from the remainder of the muscle in the ICM complex^7,8,14^. Examples of ICM trauma grade 0 to 3 following the new protocol is illustrated in Fig. 1.

In concordance with the protocol for grading the PCM^10^, the total score for the ICM was the sum of both muscle sides, ranging from 0 to 6 and categorized as: 0 = normal/no damage, 1–3 = minor damage (expect for a unilateral score of “3”,i.e. an ICM hernia, which is labelled “major”), 4–6 = major damage. Damage assessment of the ICM and PCM was conducted on all included scans by two researchers (IAA, FvdN) blinded to one another’s scores. Intraclass correlation coefficient (ICC) was calculated between these two raters to determine inter-rater reliability in ICM only. Differences in total scores between observers were discussed to reach a consensus to determine final scores for analysis.

### Statistical analysis

Statistical analysis was performed using IBM SPSS Statistics (version 28.0.1.0, SPSS Inc., Chicago, IL, USA). The intraclass correlation coefficients (ICCs) with the 95% confidence intervals (CI) between observers were calculated for the newly proposed ICM scoring system, using the scores per muscle side for both supine and upright assessment separately. ICC results were classified according to the subgroups defined by Landis and Koch^30^. To evaluate the difference in grading from supine to upright position of the ICM, the sign test was applied.

## Results

### Patient and demographics

Out of a total of 65 eligible patients, 1 patient was excluded due to insufficient MR image quality (artefact caused by a needle tip left behind after suturing a perineal tear following vaginal delivery), leaving 128 scans of 64 patients for analysis. Demographics and clinical data are presented in Table 1.

**Table 1:** This table presents the demographic and clinical information from the patients in our study. SD = standard deviation, n = number of patients affected, bmi = body mass index.

Age (years ± SD)		59 ± 11
Parity (median (range))		3 (2–4)
BMI (± SD)		27 ± 4
Cystocele (n(%))	Stage 0	0 (0)
	Stage 1	2 (3.1)
	Stage 2	30 (46.9)
	Stage 3	32 (50)
Rectocele (n(%))	Stage 0	6 (9.4)
	Stage 1	23 (35.9)
	Stage 2	34 (53.1)
	Stage 3	1 (1.6)
Uterine Prolapse (n(%))	Stage 0	24 (37.5)
	Stage 1	7 (10.9)
	Stage 2	22 (34.4)
	Stage 3	11 (17.2)
Urge urinary incontinence n(%))		45 (70.3)
Stress urinary incontinence n(%))		40 (62.5)
Pelvic pain n(%))		42 (65.6)
Fecal incontinence normal stool n(%))		14 (21.9)
Fecal incontinence liquid stool n(%))		25 (39.1)
Flatus incontinence n(%))		42 (65.6)
Vaginal bulge present n(%))		59 (92.1)
Incomplete evacuation (n(%))		38 (59.4)

## Muscle damage scoring

### PCM

During the scoring of the PCM a poor visibility of the PCM in upright was found as compared to a good visibility in supine. Since the PCM scoring protocol was established in supine and visibility in upright was poor, the PCM assessment was only done on the supine scans, and the resulting scores are presented in Table 2.

**Table 2:** This table presents the correlation between the incidence of damage in the pubococcygeus muscle (PCM) in supine and iliococcygeal muscle (ICM) in upright position. It shows the number of patients with none, minor and major damage in each muscle. N represents the number of patients affected in each category.

		PCM Supine			Total ICM damage (*n*(%))
		None (*n*(%))	Minor (*n*(%))	Major (*n*(%))	
ICM Upright	None (n(%))	0 (0)	2 (3.1)	0(0)	2 (3.1)
	Minor (n(%))	3 (4.7)	4 (6.3)	22 (34.4)	29 (45.3)
	Major (n(%))	1 (1.6)	11 (17.2)	21 (32.8)	33 (51.6)
Total PCM damage (n(%))		4 (6.3)	17 (26.6)	43 (67.2)	

### ICM

**Fig. 2:**
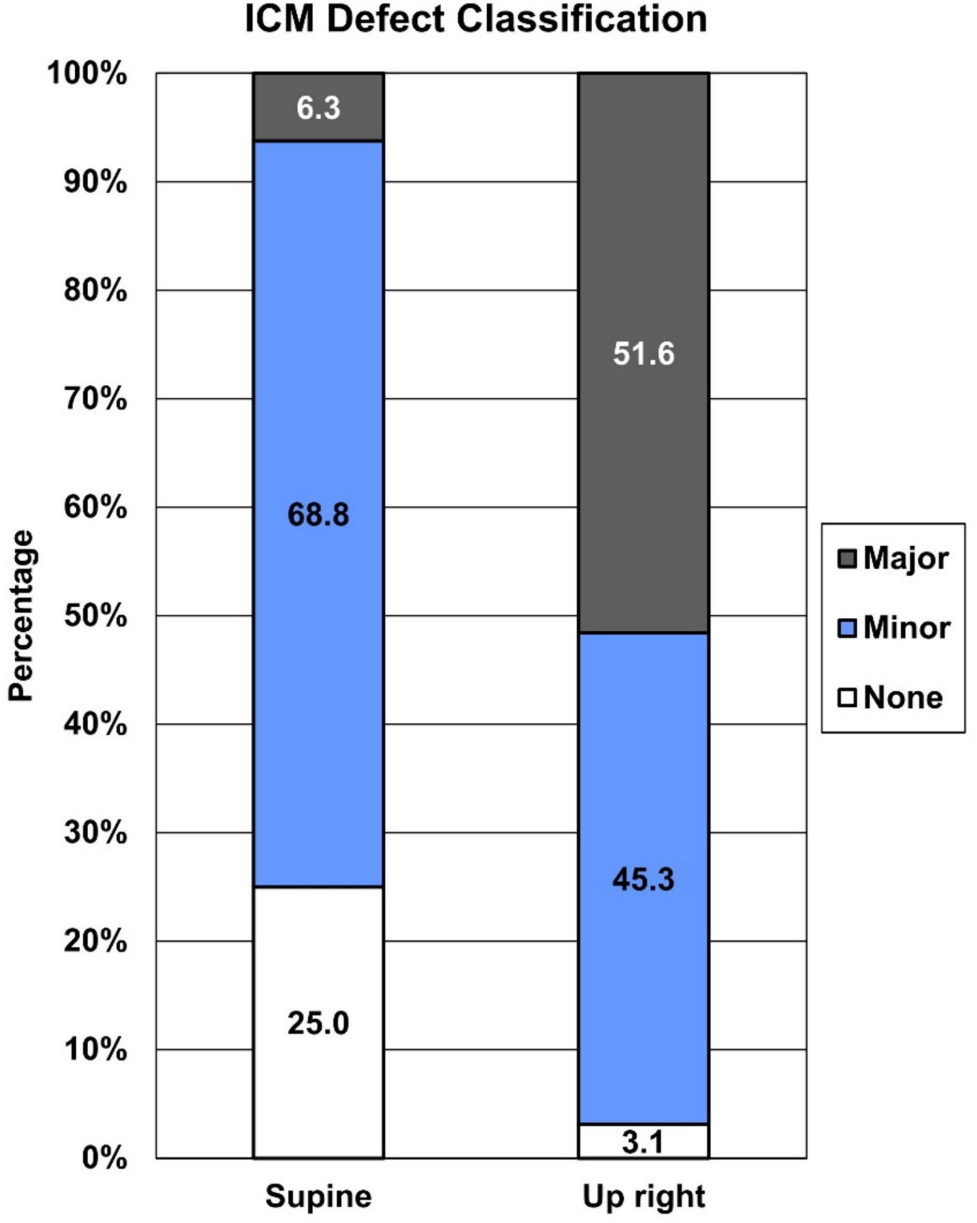
Percentages of Iliococcygeal (ICM) damage classification in none, minor and major damage for supine and upright assessment. *p* < 0.001.

ICC values (95% CI) for the ICM damage scoring were 0.68 (0.57–0.77) for supine and 0.81 (0.74–0.86) for upright assessment, indicating a substantial agreement in supine and good agreement in upright position. Differences in ICM damage (none, minor and major) in supine and upright position are visualized in Fig. 2. The sign test showed a significant difference between the supine and upright score medians (*p* < 0.001). Out of the 64 patients, 38 (59%) showed an increase in the damage scoring of the ICM, 26 (41%) patients were tied in the scoring, and none showed a decrease in the scoring rate.

**Fig. 3:**
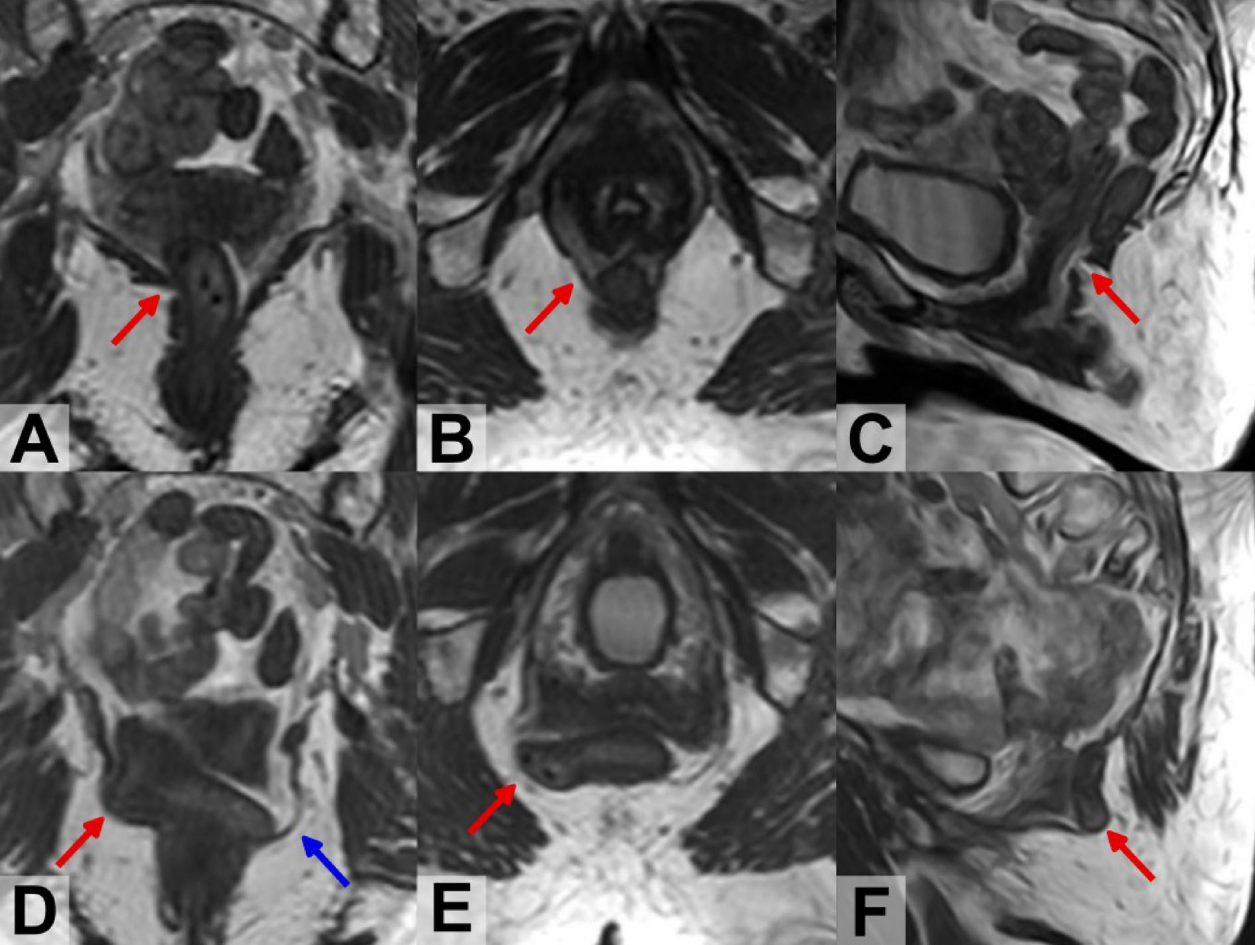
Example of differences in ICM in supine (A, B,C) and upright (D, E,F) position MR images from a 68-year-old with cystocele (POP-Q stage 3) and rectocele (POP-Q stage 2). Coronal image (**A**) in supine position at the level of the rectum showed a gap in the right iliococcygeus (red arrow) that can be seen in axial (**B**) and in sagittal (**C**), scoring as ICM minor damage. Coronal image (**D**) in up-right position at the level of the rectum showed a focal bulging in left (blue arrow) and right side of the ICM (red arrow). In axial (**E**) and sagittal (**F**) can be seen that the right damage is a herniation with rectum protrusion, scoring as major damage (red arrow).

In supine scans only two (6.3%) ICM hernias were identified (1 left and 1 right) in two patients (3.1%), while in the upright scans 27 ICM hernias were identified (15 left and 12 right) in 20 patients (31.3%); 7 patients (10.9%) had ICM hernias on both sides. A visual impression on the difference in ICM within one patient between the supine and upright scans is presented in Fig. 3. Two ICM hernias, each on a different side of the muscle, in upright position can be seen in the coronal plane (D image) and the hernia of the right is also shown in the axial (image B) and sagittal (C image) planes, while in supine there is a gap in the muscle but no herniations (A, B, C).

### Total PCM and ICM damage scores

Total damage scores (none/minor/major) in both the PCM and ICM are listed in Table 2, indicating that 34 patients (53.1%) have either major PCM or major ICM damage and 21 patients (32.8%) have major damage in both muscles, summing to a total of 85.9% patients with major damage.

## Discussion

### Main findings

LAM injury was assessed on supine and upright scans, ICM damage is severely underestimated in supine position, with 6.3% major damage vs. 51.6% in upright. PCM damage scoring was not feasible upright and is therefore based on supine scans. In our POP population we found 53.1% of women with major damage to either the PCM or ICM and 32.8% in both muscles, leading to a total of 85.9% with major damage. The new ICM assessment protocol, shows good observer variation outcomes.

In previous studies ICM damage was reported^8,9,11,12,14,26-29^ and also hernias were described (not always called hernias)^7,8,12,14,28^. However, we found no protocol for ICM damage assessment, therefore, we developed one following the principal discrimination between none, minor and major damage of the PCM protocol^10^.

In our protocol generalised ballooning and small gaps of the ICM were considered normal, score “0”. Ballooning is reported in asymptomatic nulliparous women, during Valsalva^24^, and therefore expected in upright position. Also, small gaps were reported in asymptomatic nulliparous women^25^.

Score “1” was given for ICM thinning and larger disruptions. Thinning has been reported previously^9,11,12,26^ and shown not to be associated with increased POP severity^26^, but was still considered pathological together with larger disruptions^26^. Furthermore, significantly more muscle disruptions were reported in patients compared to asymptomatic controls^31^. We observed that larger muscle disruptions often were accompanied by thinning or waviness in the muscle (‘crack phenomenon’^12^.

Score “2” and “3” were given when the ICM presented with a focal bulge, which are identified in several studies^7-9,12,14,27-29^. As Pannu et al.^8^ defined a hernia as being focal and > 10 mm, we took this definition to discriminate between a score “2” and “3”. We expect smaller bulges to have a less severe impact on the ICM’s structural integrity.

The striking difference in ICM hernias when assessing patients in supine and upright position (2 versus 20 respectively) is in line with previous studies^7,8,14^. Hernias were found during muscle straining and only one is reported in rest^8^. The reported incidence of hernias^7,8,14^ ranges from 13.5 to 15% which is lower than the 31.3% in our study. This might be due to population differences; however, we think that previous reported incidence is an underrepresentation of hernias. We hypothesize that the effect of gravity on the ICM in upright position is more severe than the effect of supine straining, in agreement with the previously observed effect that the pelvic organs descend is greater in upright position compared to supine straining^19^.

PCM damage has been studied extensively by means of MRI and US, using previously established protocols^10,18^. We conclude that PCM damage evaluation is not feasible on our upright scans. We hypothesise that gravity in supine scans helps to visualize damage by pulling the PCM from the pubic bone, enlarging the gap between these structures, while in upright position the PCM is only pulled down.

Our study assed LAM damage (supine and upright) and found a high incidence of major ICM damage. Although a correlation with symptoms is lacking, ICM damage seems to reflect significant lack in LAM support. Research and clinical assessment of the LAM have focused on PCM damage, therefore the effect of ICM damage on PFD pathofysiology is unknown. This should be investigated by comparing an asymptomatic control group to a patient population^32^, to correlate ICM damage to PFD. Furthermore, investigation of parameters contributing to ICM damage is needed, e.g. delivery, aging. This study adds to our understanding of LAM integrity and adds to recent work^19-21^ on the necessity of upright assessment when answering PFD related questions.

### Strengths and limitations

We use a low-field MRI, therefore, the image quality is less in comparison to most clinical scanners, but sufficient to classify LAM damage. A high field upright MRI scanner can provide superior image quality which might enable upright discrimination between PCM and ICM. Clinically upright MRI scanners are not widely available, making our results not immediately implementable in the clinical practice. We did not used the 3-dimensional ultrasound due to the limitation deepness where the muscles are located. In contrast, MRI provides a more comprehensive assessment of deep pelvic floor muscles.

The main strength of our study is the comparison of upright and supine imaging within one patient to identify differences in damage assessment. This study adds a new protocol for upright ICM assessment to the existing PCM protocol, offering a complete guideline for LAM damage assessment.

### Interpretation

Our study assed LAM damage (supine and upright) and found a high incidence of major ICM damage. Previous studies and clinical assessment of the LAM have mainly focused on PCM damage and defined a correlation between PCM damage and POP. However, not all POP patients are diagnosed with PCM damage. Since a full understanding of POP etiology is lacking, and ICM is an essential part of LAM support, a more detailed look into ICM integrity (e.g. sagging^33^ is indicated. This would entail the comparison of an asymptomatic control group to a patient population^32^, and correlate ICM damage to PFD. Furthermore, investigation of parameters contributing to ICM damage is needed, e.g. delivery, aging. This study adds to our understanding of LAM integrity and recent work^19,20,21^ on the necessity of upright assessment when answering PFD related questions. It is our believe that to develop effective treatment for POP, we need to fully understand the causes of POP.

## Conclusion

There is a significant difference in LAM damage assessment between supine and upright position. Supine imaging leads to an underestimation of ICM damage up to 59%, while for the PCM supine damage assessment remains superior.

## Data Availability

The datasets used and/or analysed during the current study are available from the corresponding author on reasonable request.
